# Supplementary material for: The Effect of Substrate on the Nutrient Content and Fatty Acid Composition of Edible Insects
Source: Insects. 2022 Jun 28;13(7):590. doi: 10.3390/insects13070590 (PMC9321513; doi:10.3390/insects13070590)
Supplement: Supplementary file 1 [file insects-13-00590-s001.zip › insects-1747500-supplementary.pdf]

Table S1. Macronutrients (ash, fat, protein, carbohydrate, and fiber) and calcium (Ca), phosphorous (P), and copper (Cu) content (% of dry matter, DM) of the substrates used for rearing edible insects.

Substrate	DM (%)	Ash (%)	CF, EE (%)	CP (%)	CH (%)	Fiber (%)	Ca (%)	P (%)	Cu (%)	Reference
**House cricket /*Acheta domesticus***										
Pellet	95.8						3.02	1.08		[19]
Alfalfa	18.5						1.45	0.54		
Nutrobal	97.5						20.28	0.54		
Carnivore + B Vits	99.8						35.28	0.08		
Cricket Diet Plus	94.9						12.14	0.80		
Cricket Diet	94.4						7.15	0.91		
PPF (22% prot.)	91.3	5.7	1.4	21.9	65.0	5.9				[17]
PB (19% prot.)	98.9	6.7	3.4	18.9	65.3	5.6				
BF (16% prot.)	88.7	7.9	5.2	15.8	65.6	5.3				
PPFP (18% prot.)	68.5	5.7	1.7	17.5	69.1	6.0				
PPDP (20% prot.)	89.1	5.7	2.2	19.6	66.5	6.1				
Control	89.9		4.0 [4]	17.2				0.66		[18]
HPHF	95.0		9.5 [4]	21.9				0.56		
HPLF	95.1		1.0 [4]	22.9				0.53		
LPHF	89.1		14.6 [4]	12.9				0.22		
LPLF	89.1		2.1 [4]	14.4				0.21		
**Lesser mealworm / *Alphitobius diaperinus***										
Control B-Ad	87.4		5.0	17.8						[8]
HPHS	86.5		5.5	32.7						
HPLS	95.1		5.8	39.1						
LPHS	95.7		2.3	11.9						
**Black soldier fly / *Hermetia illucens***										
Chicken feed	75.6	10.1	5.4	18.2	54.3	7.5	1.14	0.50	0.003	[29]
Brewery waste	80.2	6.5	7.5	13.5	55.3	9.0	0.52	0.46	0.015	
Food remains	70.7	8.2	7.2	20.1	42.8	6.8	0.54	0.24	0.011	
Fruits' waste	74.9	8.3	6.7	15.2	47.3	3.1	0.95	0.22	0.005	
Hen diet	92.1	13.5	4.0	17.0	49.8 [1]	15.7 [2]				[20]
Okara	18.3	4.1	17.2	39.2	7.47 [1]	32.0 [2]				
Maize distillers	94.9	5.4	11.1	29.5	17.3 [1]	36.7 [2]				
Brewer's grains	15.8	4.1	2.9	15.8	11.2 [1]	53.6 [2]				
C (control)	87.5	3.3	4.2	10.6	59.7 [1]	22.2 [2]				[21]
TMD1	87.1	2.4	4.0	11.1	68.9 [1]	13.6 [2]				
TMD2	89.5	4.1	4.4	11.2	51.1 [1]	29.2 [2]				
TMD3	88.2	4.1	4.0	13.8	56.1 [1]	22.0 [2]				
Hen diet	92.1	13.5	4.0	17.0	49.8 [1]	15.7 [2]				[26]
Okara	18.3	4.1	17.2	39.2	7.5 [1]	32.0 [2]				
Maize distillers	94.9	5.4	11.1	29.5	17.3 [1]	36.7 [2]				
Brewer's grains	15.8	4.1	2.9	15.8	11.2 [1]	53.6 [2]				
Standard diet	25	4.6	2.9	15.6	37.2 [1]	39.7 [2]				[25]
Brewer's waste	22	3.7	5.8	22.6	8.9 [1]	59.0 [2]				
Pig manure	21	13.7	1.2	26.6	0 [1]	61.2 [2]				
Semidigested grass	12	7.5	1.4	15.0	1 [1]	75.1 [2]				

VEGFRU	8.3	9.1	2.6	12.0	58.5 [3]	17.8 [2]				[24]
FRU	13.2	3.0	2.8	4.6	75.7 [3]	13.9 [2]				
WIN	35.8	10.3	7.9	11.7	13.4 [3]	56.6 [2]				
BRE	23.2	4.0	8.7	20.1	22.6 [3]	44.7 [2]				
Control	90.0		3.5 [4]	19.1				0.67		[18]
HPHF	95.0		9.5 [4]	21.9				0.56		
HPLF	95.1		1.0 [4]	22.9				0.53		
LPHF	89.1		14.6 [4]	12.9				0.22		
LPLF	89.1		2.1 [4]	14.4				0.21		
CM	93.3	20.2	2.7	15.3		35.5 [2]	0.19	0.10	0.06	[27]
KW	92.7	7.2	7.2	20.0		38.9 [2]	0.19	0.20	0.06	
SG	84.6	6.2	7.2	12.2		49.9 [2]	0.35	0.48	0.09	
T1 (SCR, *L. Buchneri*)	18.7		3.9	15.4				0.2	0.003	[46]
T2 (SCR)	18.7		3.9	15.4				0.2	-	
T3 (AF)	93.0		0.8	20.8				0.5	0.004	
Chicken feed	25.8	11.5	5.3	17.5	42.5 [1]	23.2	2.95	0.56	0.003	[28]
Digestate	24.3	29.9	6.2	24.6	0.7 [1]	38.6	1.56	1.54	0.002	
Vegetable waste	12.7	10.8	2.1	8.6	44.9 [1]	33.6	0.68	0.24	0.001	
Restaurant waste	26.2	4.5	13.9	15.7	61.8 [1]	4.1	0.14	0.24	0.000	
Control (middlings)	86.3	10.1	5.9	22.0	56.5 [5]	5.5	1.41	0.77	0.005	[30]
Protein (dried distillers' grains)	93.0	5.9	8.4	31.2	46.9 [5]	7.6	0.08	0.96	0.001	
Fibre (dried sugar beet pulp)	91.0	8.3	1.1	8.5	66.5 [5]	15.6	1.15	0.09	0.001	
**Yellow mealworm / *Tenebrio molitor***										
Pellet	95.8						3.02	1.08		[19]
Alfalfa	18.5						1.45	0.54		
Nutrobal	97.5						20.28	0.54		
Carnivore + B Vits	99.8						35.28	0.08		
Mealworm Diet Plus	95.2						8.13	0.93		
Cricket Diet	94.4						7.15	0.91		
SG (spent grains)	94.8	3.6	3.5	19.0	73.9					[31]
B (bread)	97.1	1.9	0.3	11.5	86.3					
C (cookies)	100.0	0.7	10.5	6.6	82.3					
WB		5.3	4.1	19.6		42.5 [2]				[32]
BSG		3.8	7.3	22.5		58.0 [2]				
Control 1	89.3		4.9 [4]	17.5				0.25		[18]
Control 2	89.3		4.2 [4]	17.1				0.54		
HPHF	95.0		9.5 [4]	21.9				0.56		
HPLF	95.1		1.0 [4]	22.9				0.53		
LPHF	89.1		14.6 [4]	12.9				0.22		
LPLF	89.1		2.1 [4]	14.4				0.21		
S1	90.3	0.8	1.2	13.2	84.2 [5]	0.6				[33]
S2	91.9	5.1	5.7	16.8	62.8 [5]	9.5				
S3	78.0	4.8	6.2	15.4	56.9 [5]	16.8				
S4	62.6	4.9	7.0	14.9	51.7 [5]	21.6				
S5	46.8	5.3	7.4	11.8	44.1 [5]	31.5				

Control B-Tm/Za	89.0		3.0	17.1		[8]
HPHS	86.5		5.5	32.7		
HPLS	95.1		5.8	39.1		
LPHS	95.7		2.3	11.9		
Wheat bran		5.0	10.1	17.0	67.9	[34] [6]
MSCS		6.9	12.8	3.9	76.4	
HDSM		11.6	4.8	43.2	40.5	
SDG		15.0	6.9	13.9	64.2	

CF = Crude fat, EE = Ether extract, CP = Crude protein, CH = Carbohydrates

[1] Non-fibrous carbohydrate (NFC)
[2] Neutral detergent fiber (NDF)
[3] Non-structural carbohydrates
[4] Total fatty acids (TFA)
[5] Nitrogen free extract (NFE)
[6] Analyzed before rearing of insects

Table S2. Macronutrients (ash, fat, protein, carbohydrate, and fiber) and calcium (Ca), phosphorous (P), and copper (Cu) content (% of dry matter, DM) of the edible insects.

Substrate	DM (%)	Ash (%)	CF, EE (%)	CP (%)	CH (%)	Fiber (%)	Ca (%)	P (%)	Cu (%)	Reference
**House cricket /*Acheta domesticus***										
Pellet							0.22	0.78		[19] [7]
Alfalfa							0.17	0.81		
Nutrobal							0.38	0.89		
Carnivore + B Vits							0.58	0.88		
Cricket Diet Plus							0.81	0.81		
Cricket Diet							0.67	0.81		
PPF (22% prot.)	29.9	4.6	8.9	76.2	10.2	3.7				[17]
PB (19% prot.)	29.5	4.4	19.3	71.0	5.2	6.4				
BF (16% prot.)	31.7	4.5	14.8	70.4	10.3	7.5				
PPFP (18% prot.)	40.6	2.9	43.9	48.1	5.1	4.5				
PPDP (20% prot.)	29.3	4.5	12.9	75.8	6.8	6.6				
Control	24.1		17.4 [5]	57.8				0.89		[18]
HPHF	25.7		20.8 [5]	59.2				0.85		
HPLF	24.0		20.8 [5]	-				-		
LPHF	25.1		-	-				-		
LPLF	24.8		-	-				-		
**Lesser mealworm / *Alphitobius diaperinus***										
Control B-Ad	33.3		24.3	61.7						[8]
HPHS	31.8		21.8	64.3						
HPLS	30.0		18.1	65.0						
LPHS	33.3		13.4	-						
**Black soldier fly / *Hermetia illucens***										
Chicken feed	76.6	4.1	31.3	48.9	56.9	9.2	1.29	0.46	0.003	[29] [9]
Brewery waste	76.6	11.2	33.2	44.5	51.8	10.3	0.52	0.21	0.002	
Food remains	82.8	9.5	34.3	33.0	44.0	5.2	0.52	0.18	0.003	
Fruits' waste	72.3	13.9	34.1	38.3	47.0	5.2	0.95	0.41	0.006	
Hen diet	38.9	11.7	25.1	52.8						[20] [10]
Okara	37.4	5.9	31.2	51.2						
Maize distillers	38.5	4.9	29.9	53.4						
Brewer's grains	36.5	7.4	23.2	54.1						
C (control)	33.3	10.1	33.0	34.0						[21] [9]
TMD1	32.2	5.5	46.9	22.2						
TMD2	34.4	11.0	31.9	34.7						
TMD3	33.2	8.9	32.5	34.2						
Hen diet	38.9	13.5	26.0	48.2						[26] [10]
Okara	37.4	5.8	32.1	49.8						
Maize distillers	38.4	4.6	30.3	51.7						
Brewer's grains	35.6	7.7	25.1	52.5						
Standard diet		4.0	36.4	50.6						[25] [10]
Brewer's waste		5.7	33.7	49.9						
Pig manure		24.0	17.5	45.9						
Semidigested grass		29.6	5.5	46.4						

VEGFRU	22.0	13.0	26.3	41.9 [1]<br>31.3 [2]		17.1 [4]				[24] [10]
FRU	28.3	7.2	40.7	30.8 [1]<br>23.0 [2]		19.8 [4]				
WIN	26.5	14.6	32.2	34.4 [1]<br>25.7 [2]		17.7 [4]				
BRE	29.1	7.3	29.9	53.0 [1]<br>39.6 [2]		8.7 [4]				
Control	33.9		25.4 [5]	43.8				0.97		[18] [10]
HPHF	32.9		24.7 [5]	46.3				0.85		
HPLF	35.6		25.5 [5]	43.5				0.86		
LPHF	35.1		28.0 [5]	38.8				0.67		
LPLF	35.3		33.5 [5]	38.3				0.64		
CM	80.7	9.3	30.1	41.1		21.9 [4]	0.32	0.39	0.04	[27] [10]
KW	87.7	9.6	34.3	33.0		20.4 [4]	0.2	0.41	0.02	
SG	83.1	11.6	31	41.3		28.6 [4]	0.17	0.46	0.05	
T1 (SCR, *L. Buchneri*)			30.0	55.3					0.0006	[46] [10]
T2 (SCR)			26.1	52.9					0.0006	
T3 (AF)			24.3	50.4					0.0008	
Chicken feed	38.7	10.0	33.6	41.2			2.87	0.50	0.001	[28] [9]
Digestate	38.6	19.7	21.8	42.2			6.62	0.44	0.001	
Vegetable waste	41.0	9.6	37.1	39.9			2.87	0.40	0.001	
Restaurant waste	38.1	2.7	38.6	43.1			0.12	0.41	0.001	
Control (middlings)	30.2	13.5	30.8	37.2			3.94	0.89	0.003	[30] [10]
Protein (dried distillers' grains)	30.2	4.8	38.6	44.6			0.54	0.87	0.001	
Fibre (dried sugar beet pulp)	17.9	22.9	3.4	52.3			6.16	1.32	0.001	
**Yellow mealworm / *Tenebrio molitor***										
Pellet							0.22	0.79		[19] [7]
Alfalfa							0.13	0.71		
Nutrobal							0.57	0.75		
Carnivore + B Vits							1.05	0.74		
Mealworm Diet Plus							0.56	0.78		
Cricket Diet							0.50	0.76		
SG (spent grains)	33.3	3.4	19.4	51.3 [1]<br>39.1 [3]	25.2 [1]<br>37.6 [3]					[31]
B (bread)	32.6	3.1	45.4	42.3 [1]<br>32.2 [3]	8.3 [1]<br>18.6 [3]					
C (cookies)	35.6	2.7	50.0	37.3 [1]<br>28.4 [3]	9.8 [1]<br>18.8 [3]					
WB	36.9	1.3	12.4	13.4						[32] [8]
BSG	33.7	1.3	6.4	14.8						
Control 1	39.8		27.0 [5]	52.4				0.97		[18]
Control 2	39.2		30.9 [5]	49.2				0.77		
HPHF	41.5		26.5 [5]	53.6				0.89		
HPLF	36.7		23.0 [5]	53.5				0.88		
LPHF	37.2		26.8 [5]	44.4				0.88		
LPLF	38.2		28.5 [5]	47.5				0.82		

S1	35.3	3.5	40.1	37.8	12.7 [6]	6.0	[33]
S2	39.5	3.9	34.0	50.1	4.9 [6]	7.1	
S3	38.1	3.9	32.1	47.6	8.1 [6]	8.3	
S4	36.7	3.9	35.3	39.4	13.4 [6]	8.0	
S5	31.5	4.6	36.1	38.1	11.1 [6]	10.2	
Control B-Tm/Za	27.3		25.0	45.1			[8]
HPHS	33.4		26.3	48.6			
HPLS	29.4		27.6	47.5			
LPHS	33.3		18.9	46.9			
Wheat bran		8.1	16.9	68.9	6.0		[34]
MSCS		5.9	6.1	76.3	11.7		
HDSM		6.6	8.1	74.4	10.8		
SDG		7.7	12.0	70.1	10.2		

CF = Crude fat, EE = Ether extract, CP = Crude protein, CH = Carbohydrates, - = Values are not available

[1] Conversion factor 6.25 for crude protein
[2] Conversion factor 4.67 for crude protein
[3] Conversion factor 4.76 for crude protein
[4] Neutral detergent fiber (NDF)
[5] Total fatty acids (TFA)
[6] Nitrogen free extract (NFE)
[7] After 72 h of rearing
[8] On a fresh weight basis (%FW)
[9] prepupae
[10] larvae
